# Cardiac structure and function recovery in acromegaly after treatment: insights from cardiac magnetic resonance imaging

**DOI:** 10.3389/fendo.2025.1630037

**Published:** 2025-07-31

**Authors:** Yu Chen, Yangjie Li, Yu Tang, Jing Li, Yerong Yu, Huiwen Tan, Bowen Cai, Shu Jiang, Wei Wang, Songping Zheng, Peizhi Zhou, Yi Wei, Jiayu Sun, Yucheng Chen, Jianwei Li

**Affiliations:** ^1^ Department of Endocrinology and Metabolism, West China Hospital, Sichuan University, Chengdu, Sichuan, China; ^2^ Department of Cardiology, West China Hospital, Sichuan University, Chengdu, Sichuan, China; ^3^ Department of Neurosurgery, West China Hospital, Sichuan University, Chengdu, Sichuan, China; ^4^ Department of Radiology, West China Hospital, Sichuan University, Chengdu, Sichuan, China

**Keywords:** acromegaly, cardiac magnetic resonance imaging, heart structure, cardiac function, myocardial lesions

## Abstract

**Purpose:**

Cardiac magnetic resonance imaging (CMRI) provides a detailed method for understanding the specific cardiovascular alterations associated with acromegaly. This study aimed to evaluate the impact of personalized treatment on cardiac structure, function, and myocardial tissue characteristics using CMRI, and to assess the effects of biochemical remission on improving cardiovascular complications in acromegaly patients.

**Method:**

Thirty-nine acromegaly patients were enrolled from July 2020 to February 2023 at West China Hospital of Sichuan University. Comprehensive cardiac assessments were conducted using a 3.0 T MRI scanner at baseline and one year after individualized treatment.

**Results:**

Among the 30 patients who completed both baseline and one-year follow-up CMRI examinations, significant reductions in left ventricular (LV) wall thickness and end-diastolic diameter were observed compared to baseline (both *P* < 0.05). Left ventricular mass (LVM) and mass index (LVMi) also significantly decreased (LVM: 90.17 ± 25.86 g *vs.* 101.18 ± 26.10 g, *P*=0.007; LVMi: 50.01 ± 12.56 g/m^2^ *vs.* 56.20 ± 13.01 g/m², *P*=0.008). Additionally, T2 values showed a significant reduction following individualized treatment (basal T2: 39.12 ± 2.82 ms *vs.* 42.91 ± 4.38 ms, *P*<0.001; apical T2: 41.96 ± 3.87 ms *vs.* 45.13 ± 4.87 ms, *P*=0.021). Compared to healthy controls, patients who achieved biochemical remission exhibited increased LV inferior septal thickness and elevated extracellular volume (ECV) values. However, T2 value in the basal layer of the LV was significantly lower in the remission group compared to healthy controls.

**Conclusion:**

Patients with acromegaly exhibited LV hypertrophy, enlargement, myocardial fibrosis and impaired systolic function assessed by CMRI compared with healthy controls. Individualized treatment led to partial reversed of these abnormalities, particularly in those who achieved biochemical remission.

## 1 Introduction

Acromegaly is a relatively rare chronic endocrine disorder primarily caused by growth hormone pituitary adenomas (GHPA) (1). Elevated levels of growth hormone (GH) lead to the excessive production of insulin-like growth factor-1 (IGF-1), resulting in typical physical features such as a prominent brow, thickened lips, a protruding lower jaw, and enlargement of the extremities (1). Beyond the characteristic morphological changes, acromegaly adversely affects various organ systems, including the cardiovascular, respiratory, musculoskeletal, and metabolic systems, and caused complications such as hypertension, myocardial hypertrophy, heart failure, obstructive sleep apnea-hypoventilation syndrome (OSAHS), musculoskeletal disorders, and diabetes (2).

Compared to the general population, acromegaly patients with untreated or inadequately treated have a 61% higher risk of mortality, primarily due to cardiovascular-related complications (3). Notably, cardiovascular issues in acromegaly include hypertension, myocardial disease, valvular heart disease, and arrhythmias (4), leading to a threefold rise in hospitalization risk and a significant annual healthcare cost escalation (5). Accurate and comprehensive cardiac assessment is crucial for the diagnosis and treatment of cardiac complications in acromegaly. Despite the widely use of echocardiography in the cardiac evaluation of acromegaly patients, its accuracy and repeatability are compromised due to its substantial reliance on operator experience and appropriate echocardiographic window positioning (6). Recent researches have revealed that cardiac complications in acromegaly, such as reduced myocardial perfusion, myocardial edema, myocardial fibrosis, and local myocardial dyssynchrony, significantly impact patients’ quality of life and even survival. However, echocardiography faces challenges in accurately assessing the above-mentioned conditions (7).

Cardiac MRI incorporates various techniques such as cine sequences, mapping, and late gadolinium enhancement (LGE). Its clinical application is becoming increasingly widespread (8). The LGE technique in Cardiac MRI is currently considered the “gold standard” for non-invasive assessment of focal myocardial fibrosis (9). T1 mapping technology can quantitatively assess myocardial tissue fibrosis by directly measuring T1 values. Through the incorporation of T1 times and hematocrit levels, extracellular volume (ECV) can be obtained, offering distinctive advantages in evaluating diffuse myocardial fibrosis (10). T2 mapping technology employs T2 relaxation times to quantitatively analyze myocardial tissue water content, which can be used for assessing myocardial edema in patients with acromegaly.

To date, research on the assessment of cardiac involvement in acromegaly using Cardiac MRI remains limited, with inconsistent findings across studies. Bogazzi et al. used Cardiac MRI in 14 newly diagnosed acromegaly patients before and after treatment with somatostatin analogues (SSAs) (11), and found a significant improvement in left ventricular mass index (LVMi) following SSAs treatment. However, research by Brazilian scholars indicated that octreotide failed to improve cardiac structural or functional parameters in acromegaly patients (12). In 2020, Xing et al. utilized Cardiac MRI in acromegaly patients after transsphenoidal surgery (TSS) (13) and demonstrated a significant improvement in left ventricular anterior wall hypertrophy after operation, regardless of achieving endocrine remission. Moreover, the patients who achieve endocrine remission had greater improvement in left ventricular anterior wall thickness than those who did not achieve. Previous exploration of cardiac complications in acromegaly using Cardiac MRI technology has provided valuable insights. However, further studies in a larger cohort, and a more precise assessment of cardiac status in acromegaly is remain imperative, which will contribute to a more comprehensive understanding of the intricate interplay between acromegalic pathology and cardiac manifestations.

In this prospective cohort study, we employed Cardiac MRI techniques to systematically assess alterations in cardiac structure, function, and myocardial tissue characteristics among acromegaly patients before and after personalized treatment over a one-year period. Furthermore, we investigated the potential reversal of cardiac structural or functional changes in acromegaly patients upon achieving biochemical remission.

## 2 Materials and methods

### 2.1 Patient population

From July 2020 to February 2023, patients newly diagnosed with acromegaly at the Department of Endocrinology and Metabolism in a tertiary hospital were consecutively enrolled in the study. The inclusion criteria were as follows (1): patients who were newly diagnosed with acromegaly based on the following criteria (14): a) GH nadir following an oral glucose tolerance test (OGTT) ≥1 ng/ml or IGF-1 levels exceeding the age- and gender-matched reference range; b) presentation of typical clinical manifestations of acromegaly; c) identification of a pituitary adenoma through sellar magnetic resonance imaging (MRI) (2); patients aged 18 years and older (3); patients with willingness to participate in and tolerate Cardiac MRI examination. The exclusion criteria were as follows: (1) individuals with documented organic heart conditions, including but not limited to rheumatic heart disease or coronary artery disease; (2) patients with malignant tumors or diagnosed with malignant tumors or other debilitating conditions impeding regular follow-up; (3) individuals unable to actively participate in or tolerate Cardiac MRI examinations; or (4) patients exhibiting with study requirements. This study was approved by the institutional review board [No. 2020 (335)] with written informed consent obtained from all participants.

### 2.2 Study design and follow-up

Patients diagnosed with acromegaly underwent comprehensive clinical evaluation, including pre-treatment Cardiac MRI. Pertinent clinical parameters, such as gender, age, body mass index (BMI), and disease duration, alongside hormonal markers (random GH, GH nadir and IGF-1) were meticulously documented. According to international guidelines(1), the multidisciplinary team for pituitary tumors, composed of neurosurgery, endocrinology and metabolism, radiology, and other departments, formulates different treatment plans based on the characteristics of the patients, with subsequent outpatient or telephonic follow-ups scheduled at three-month intervals post-treatment. Subsequent to the initial intervention, a follow-up Cardiac MRI examination was one year later, coupled with hormonal assessments throughout the follow-up period. Biochemical remission was defined as age-gender normalized IGF-1 or a GH nadir of <1ng/ml (14–16). The IGF-1 reference range employed in this investigation was derived from Zhu’s study (17).

### 2.3 Control group selection and matching methods

The healthy control group consisted of healthy volunteers recruited during the same period. All participants underwent cardiac magnetic resonance imaging. In this study, a 1:1 matching method was employed to select healthy volunteers for the control group who were matched to patients in the acromegaly group based on age, gender, and height (same gender, age within a 3-year range, and height within a 5 cm range). Due to difficulties in height matching for some acromegaly patients, a total of 32 pairs were matched using the 1:1 ratio. There were no significant differences in age, gender, or height between the two groups, demonstrating good comparability (as shown in **Table 1**).

**Table 1:** Comparison of general characteristics between the acromegaly group and the healthy control group.

Parameters	Acromegaly group n=32	Healthy control group n=32	P
Age, years	44.25 4.13.09	43.71 3.12.73	0.124
Male, n(%)	8 (25.0%)	8 (25.0%)	1
Height, cm	162.88 626.98	162.28 628.07	0.489

Continuous variables with a normal distribution are presented as mean ± standard deviation. Categorical variables are presented as percentages.

### 2.4 Hormone assays

The glucose growth hormone suppression test is performed after the patient has fasted in the early morning and orally ingested 75g of glucose. Subsequent measurements of GH levels are taken at intervals of 0, 30, 60, 90, and 120 minutes. For other hormone analyses, fasting blood samples are collected in the early morning. All hormonal values are quantified utilizing the electrochemiluminescence method.

### 2.5 Cardiac MRI image acquisition

This study utilized a 3.0T magnetic resonance imaging (MRI) machine (MAGNETOM Trio or Skyra, Siemens Healthcare) for scanning. A steady-state free precession (SSFP) cine sequence was used to acquire continuous short-axis cine images of the left ventricle, as well as standard two-chamber, three-chamber, and four-chamber cine images in the long-axis direction. Scan parameters were as follows: field of view: 280mm x 340mm; repetition time/echo time: 3.4 ms/1.3 ms; matrix size: 256mm x 144mm; slice thickness: 8mm; flip angle: 50°; temporal resolution: 42ms.

T1 mapping images were performed using modified Look-Locker inversion recovery sequence before and 10 minutes after contrast injection, which included scans of the left ventricular short-axis basal, mid-ventricular, and apical levels. The scan parameters were as follows: field of view: 360mm x 360mm; repetition time/echo time: 3.75ms/1.67ms; matrix size: 224mm x 224mm; slice thickness: 8mm; flip angle: 35°. Delayed enhancement short-axis scans were acquired 10–15 minutes after contrast agent injection using a phase-sensitive inversion recovery sequence with the following parameters: field of view: 260mm x 340mm; matrix size: 116mm x 192mm; slice thickness: 8mm; repetition time: 700ms; echo time: 2.0ms; post-inversion delay time: 300-380ms; flip angle: 20°. T2 mapping was conducted using a T2-prepared single-shot steady-state free precession sequence before contrast injection. Parameters: gradient echo; TR/TE = 240 ms/1.0 ms; FA = 12°; FOV = 280 × 360; matrix = 116 × 192; slice thickness = 8 mm; and T2 preparation pulses with 0-, 30-, and 55-ms echo times.

### 2.6 Cardiac MRI parameter extraction

After image acquisition, dedicated MRI post-processing software (Medis, Amsterdam, The Netherlands) was used for measurements and analysis.

**Figure 1:**
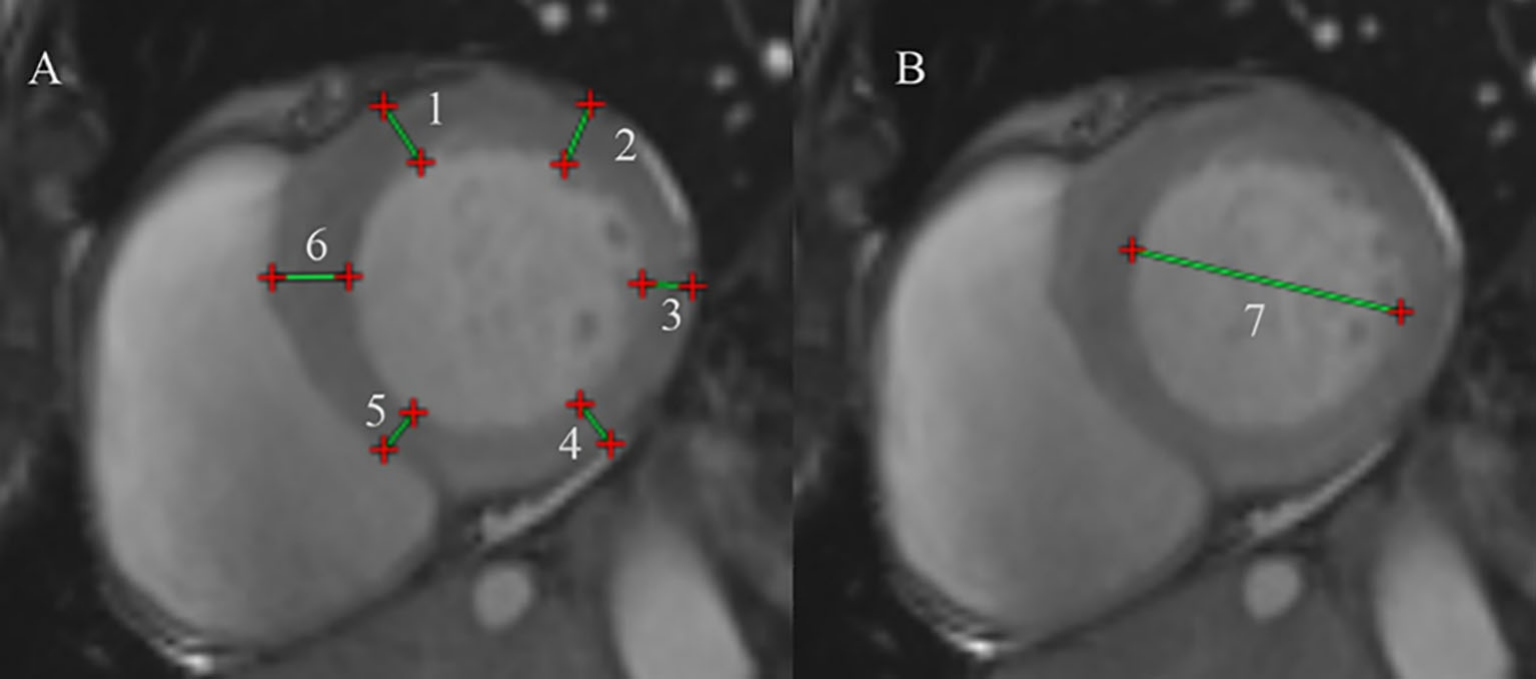
Protocol for CMR contouring of the ventricles root in acromegaly patients. **(A)** Measure the left ventricular wall thickness at the basal segment of the left ventricle. (1) Left ventricular (LV) anterior wall thickness, (2) LV lateral wall thickness, (3) LV posterior wall thickness, (4) LV inferior wall thickness, (5) inferior septum, (6) anterior septum. **(B)** Measure the left ventricular end-diastolic diameter at end-diastole. (7) The left ventricular end-diastolic diameter.

1. Cardiac Structure Measurements: In the short-axis basal plane of the left ventricle, measurements were taken to comprehensively assess the left ventricular wall thickness. This included the left ventricular anterior wall (LVAW), left ventricular lateral wall (LVLW), left ventricular posterior wall (LVPW), left ventricular inferior wall (LVIW), inferior septum (IS), and anterior septum (AS) (**Figure 1**). The left ventricular end-diastolic diameter (LVEDD) was measured at the end of diastole to evaluate left ventricular enlargement (**Figure 1**).
2. Cardiac Function Measurements: The biventricular function, volume, and mass was analyzed in accordance with the Society of Cardiovascular Magnetic Resonance post-processing guideline (18). This includes the measurement of left ventricular end-diastolic volume (LVEDV), left ventricular end-systolic volume (LVESV), right ventricular end-diastolic volume (RVEDV), and right ventricular end-systolic volume (RVESV). Left ventricular ejection fraction (LVEF) is calculated as (LVEDV - LVESV)/LVEDV to assess left ventricular systolic function, and right ventricular ejection fraction (RVEF) is obtained as (RVEDV - RVESV)/RVEDV to evaluate right ventricular systolic function.
3. Left Ventricular Hypertrophy Measurement: The degree of left ventricular hypertrophy was assessed by multiplying myocardial volume by myocardial specific gravity (1.05 g/cm³) to obtain left ventricular mass (LVM) (19). Left ventricular mass index (LVMi) is standardized based on body surface area (BSA) to assess left ventricular hypertrophy. BSA was calculated using the Dubois formula (20): BSA (m²) = 0.007184 × height (m)^0.725^ × weight (kg)^0.725^.
4. Myocardial Fibrosis Measurement: LGE was deemed present when the area of enhancement could be seen in two phase-encoding directions and two orthogonal views. Changes in extracellular volume (ECV) typically represent the development of diffuse fibrosis. Using the patient’s hematocrit (HCT) result, the extracellular volume (ECV) was calculated as follows (10): ECV = (1 - HCT) * (Myocardial ΔR1/Blood pool ΔR1); Myocardial ΔR1 = 1/Myocardial post-contrast T1 - 1/Myocardial pre-contrast T1, Blood pool ΔR1 = 1/Blood pool post-contrast T1 - 1/Blood pool pre-contrast T1.
5. Myocardial Edema Measurement: Images were imported into Medis software, and T2-mapping images for the left ventricular basal, mid-ventricular, and apical segments were selected sequentially. The myocardium was manually delineated, and after manual adjustment for matching, the software automatically calculated the T2 values.

### 2.7 Statistical analysis

SPSS (IBM SPSS Statistics, version 24.0, USA) was used to analyze the data. GraphPad Prism (GraphPad Software, version 9.0, USA) was used to generate bar charts. The normality of continuous data was assessed using the Shapiro-Wilk test. Normally distributed continuous data are presented as means ± SDs, while non-normally distributed continuous data are expressed as median (lower quartile, upper quartile). Count data are expressed as proportions or percentages. Independent sample t-test was used for the comparison of two groups with normally distributed data, and paired data following a normal distribution were analyzed using the paired t-test. For skewed data, the Mann-Whitney U test was applied for comparisons between two groups, and the paired Wilcoxon test was used for paired skewed data. Chi-squared test was used for comparisons involving rates or proportions. Statistical significance was defined as p<0.05.

## 3 Results

### 3.1 Characterization of the study population

A total of 39 patients with acromegaly, including 14 males and 25 females, were included (**Table 2**). Within this cohort, 25 patients (64.1%) received monotherapy, including 23 patients treated with transsphenoidal surgery (TSS), and 2 with somatostatin receptor ligands (SRLs). One patient was treated with octreotide due to poor cardiac function, which posed a high risk for surgical anesthesia, and another patient opted for octreotide therapy due to a refusal to undergo surgery. Fourteen patients (35.9%) underwent two or more treatment modalities, including 5 patients treated with TSS combined with SRLs, 1 patient with TSS and cabergoline, 6 patients with gamma knife and SRLs, and 2 patients with combination of three treatment methods.

**Table 2:** Clinical characteristics of 39 acromegaly patients at baseline.

Clinical characteristics	Acromegaly patients, n = 39
Age, years	43.54 ± 12.99
Male, n (%)	14 (35.9%)
Disease duration, years	10 (3, 18)
Body mass index, kg/m^2^	25.75 ± 3.90
Fasting growth hormone, ng/ml^a^	14.60 (8.00, 37.20)
Growth hormone nadir after the OGTT, ng/ml	14.94 (5.45, 28.63)
insulin-like growth factor 1, ng/ml	495.52 ± 168.49
anterior pituitary insufficiency, n (%)	21 (53.8%)
Microadenoma, n (%)^b^	3 (8.3%)
Macroadenoma, n (%)^b^	32 (88.9%)
Giant adenoma, n (%)^b^	1 (2.8%)
TSS, n (%)	23 (59.0%)
SRLs, n (%)	2 (5.1%)
TSS+SRLs, n (%)	5 (12.8%)
TSS+carbergoline, n (%)	1 (2.6%)
SRLs+gamma knife, n (%)	6 (15.4%)
TSS+SRLs+ gamma knife, n (%)	2 (5.1%)
Diabetes, n (%)	9 (23.1%)
Prediabetes, n (%)	4 (10.3%)
Hypertension, n (%)	18 (46.2%)
Hyperlipidemia, n (%)	9 (23.1%)
Anterior pituitary insufficiency, n (%)	21 (53.8%)
Myocardial late gadolinium enhancement, n (%)	7 (17.9%)

Continuous variables with a normal distribution are presented as mean ± standard deviation, while those with a non-normal distribution are expressed as median (interquartile range). Categorical variables are presented as percentages. OGTT, oral glucose tolerance test; TSS, transsphenoidal surgery; SRLs, somatostatin receptor ligands. ^a^The reference range for random growth hormone is 0.13-9.88 ng/ml. ^b^due to the unavailability of pre-treatment imaging data for three patients from external sources, the maximum diameter of pituitary tumors could not be accurately determined. Therefore, this study only included the analysis of pituitary tumor size in 36 patients.

Approximately one-third of acromegaly patients exhibited abnormal blood glucose levels, 46.2% with hypertension and 23.1% with hyperlipidemia, respectively when enrolled in the study. Anterior pituitary insufficiency was diagnosed in 21 patients before surgery, and 7 acromegaly patients were positive for LGE. Thirty patients (11 males and 19 females) completed the one-year follow-up. Nine did not finish the follow-up because this study was conducted during the COVID-19 pandemic, six patients chose to seek medical care at local hospitals. Additionally, three patients lacked awareness of the cardiac complications associated with acromegaly. In our study, 63.3% (19/30) of patients with acromegaly achieved biochemical remission after 1 year of treatment. Posttreatment clinical characteristics were compared with the baseline characteristics. Significant reductions in posttreatment GH and IGF-1 levels were observed, along with improvements in anterior pituitary function.

### 3.2 Comparison of cardiac MRI parameters between the acromegaly group and the healthy control group

Due to challenges in matching the height and weight of some acromegaly patients, 32 pairs were matched using the 1:1 ratio. Firstly, a comparison of cardiac structural and functional parameters between the two groups (**Table 3**) revealed that acromegaly group had higher thicknesses of the left ventricular lateral wall, posterior wall, inferior wall, inferior septum, and anterior septum than the control group, with the most pronounced difference observed in the left ventricular inferior septum (9.08 ± 1.70 *vs.* 5.76 ± 1.40mm, *P*<0.001). The left ventricular mass (LVM) and left ventricular mass index (LVMi) were also significantly greater in the acromegaly group than in the control group. Furthermore, the left ventricular end-diastolic diameter in the acromegaly patients was greater than that in the healthy controls (51.58 ± 7.07 *vs.* 46.05 6.5.12mm, *P*=0.003). The acromegaly group had lower left ventricular ejection fraction (57.72 ± 10.04 *vs.* 62.49 ± 4.91%, *P*=0.034), but similar right ventricular ejection fraction compared with the control group. Additionally, a comparison of myocardial parameters between the groups (**Table 3**) revealed that the extracellular volume (ECV) values for all myocardial layers in acromegaly patients were higher than those in the control group. There were no significant differences in the T2 values of the myocardial segments between the groups, but a trend of higher T2 values in the myocardial segments in the acromegaly group compared to that in the control group.

**Table 3:** Comparison of cardiac magnetic resonance imaging parameters between the acromegaly group and the healthy control group.

Parameters	Acromegaly group n=32	Healthy control group n=32	t	P
Cardiovascular wall thickness and ventricular mass				
LV anterior wall, mm	7.40 ± 1.75	6.62 ± 1.57	1.949	0.061
LV lateral wall, mm	8.24 ± 1.63	6.36 ± 1.02	5.477	<0.001
LV posterior wall, mm	8.70 ± 2.30	7.02 ± 1.37	2.703	0.011
LV inferior wall, mm	8.33 ± 2.43	6.33 ± 1.14	3.805	0.001
inferior septum, mm	9.08 ± 1.70	5.76 ± 1.40	8.611	<0.001
anterior septum, mm	10.52 ± 2.93	7.97 ± 1.44	4.380	<0.001
LVM(g)	96.53 ± 24.16	70.59 ± 19.34	3.853	0.001
LVMi(g/m^2^)	56.10 ± 12.98	41.40 ± 10.13	3.859	0.001
LV end-diastolic diameter, mm	51.58 ± 7.07	46.05 ± 5.12	3.257	0.003
Ventricular volume and systolic function				
Index LV end-diastolic volume, ml/m^2^	91.35 ± 35.40	75.81 ± 14.68	1.877	0.071
Index LV end-systolic volume, ml/m^2^	41.40 ± 33.60	28.79 ± 7.94	1.781	0.086
Index RV end-diastolic volume, ml/m^2^	84.16 ± 17.55	69.23 ± 18.98	2.919	0.007
Index RV end-systolic volume, ml/m^2^	39.11 ± 18.22	32.05 ± 11.06	1.645	0.111
LVEF(%)	57.72 ± 10.04	62.49 ± 4.91	-2.233	0.034
RVEF(%)	56.77 ± 10.34	54.03 ± 7.55	1.262	0.217
Myocardial Fibrosis				
Basal native T1, ms	1238.47 ± 42.84	1206.07 ± 50.52	2.619	0.015
Basal post T1, ms	573.39 ± 58.32	490.85 ± 41.01	6.015	<0.001
Basal ECV, %	29.59 ± 3.58	26.40 ± 2.73	4.220	<0.001
Middle native T1, ms	1232.29 ± 48.95	1205.94 ± 41.28	2.134	0.041
Middle post T1, ms	565.88 ± 56.20	486.45 ± 40.28	6.099	<0.001
Middle ECV, %	31.40 ± 4.32	27.15 ± 2.93	4.812	<0.001
Apical native T1, ms	1249.88 ± 87.05	1239.00 ± 74.17	0.495	0.624
Apical post T1, ms	557.14 ± 50.33	468.77 ± 45.25	7.095	<0.001
Apical ECV, %	33.23 ± 4.88	30.40 ± 4.16	2.363	0.025
Myocardial Edema				
Basal T2, ms	40.36 ± 4.05	40.12 ± 9.32	-0.034	0.973
Middle T2, ms	40.36 ± 4.05	40.02 ± 9.32	-0.877	0.389
Apical T2, ms	42.53 ± 4.44	41.15 ± 5.80	-1.092	0.285

Continuous variables with a normal distribution are presented as mean ± standard deviation. LV, left ventricular; RV, right ventricular; LVM, left ventricular mass; LVMi, left ventricular massindex; LVEF, left ventricular ejection fraction; RVEF, right ventricular ejection fraction; ECV, extracellular volume; P < 0.05, P < 0.01 and P<0.001 indicate statistically significant differences between the acromegaly group and the healthy control group.

### 3.3 Comparison of cardiac MRI parameters in patients with acromegaly before and after treatment

A total of 30 completed one-year follow-up. The comparison of the baseline clinical characteristics between acromegaly patients who completed follow-up and those who did not revealed that, on average, the patients who did not complete follow-up were older and had smaller tumors before treatment (see **Table 4**). A comparative analysis of cardiac structure and function parameters was performed for these 30 patients (**Table 5**). There were significant decrease in anterior wall, lateral wall, posterior wall, inferior wall, inferior septum, and anterior septum of the left ventricle after treatment. The most notable reductions were observed in the thickness of the left ventricular anterior wall (7.45 ± 1.71 *vs.* 6.65 ± 1.91mm, *P*<0.001), inferior septum (9.37 ± 1.85 *vs.* 8.08 ± 1.81mm, *P*<0.001), and anterior septum (10.48 ± 2.36 *vs.* 8.84 ± 1.87mm, *P*<0.001). Furthermore, the left ventricular end-diastolic diameter decreased post-treatment (52.67 ± 7.23 *vs.* 50.78 ± 6.94mm, *P*=0.017). The results indicated a significant decrease in both left ventricular mass (101.18 ± 26.10 *vs.* 90.17 ± 25.86g, *P*=0.007) and left ventricular mass index (56.20 ± 13.01 *vs.* 50.01 ± 12.56g/m^2^, *P*=0.008) after treatment. In contrast, no improvement was found in LVEF and RVEF.

**Table 4:** Comparison of the baseline clinical characteristics between acromegaly patients who completed follow-up and those who did not completed follow-up.

Clinical characteristics	Completed follow-up n=30	Not-completed follow-up n=9	t/z	P
Age, years	41.17 ± 11.99	51.44 ± 13.74	-2.183	0.035
Male, n(%)	11 (37%)	3 (33.3%)	/	0.855
Tumor size, cm	2.07 ± 0.95	1.47 ± 0.35	2.700	0.012
Disease duration, months	112.60 ± 89.68	159.00 ± 95.72	-1.341	0.188
Body mass index, kg/m^2^	25.94 ± 3.56	25.10 ± 5.08	0.564	0.576
Growth hormone nadir after the OGTT, ng/ml	10.84 (13.66, 33.89)	17.20 (7.67, 27.61)	-0.251	0.802
insulin-like growth factor 1, ng/ml	509.91 ± 158.62	449.13 ± 200.16	0.944	0.351
anterior pituitary insufficiency, n(%)	16 (53.3%)	5 (55.6%)	/	0.907

Continuous variables with a normal distribution are presented as mean ± standard deviation, while those with a non-normal distribution are expressed as median (interquartile range). Categorical variables are presented as percentages. OGTT, oral glucose tolerance test; P < 0.05 indicate statistically significant differences between acromegaly patients who completed follow-up and those who did.

**Table 5:** Comparison of cardiac structure and functional parameters in patients with acromegaly before and after 1 year of treatment.

Parameters	Baseline n=30	Posttreatment n=30	t	P
Cardiovascular wall thickness and ventricular mass				
LV anterior wall, mm	7.45 ± 1.71	6.65 ± 1.91	4.303	<0.001
LV lateral wall, mm	8.15 ± 1.73	7.46 ± 1.77	2.948	0.006
LV posterior wall, mm	8.81 ± 2.45	7.61 ± 2.00	3.322	0.002
LV inferior wall, mm	8.95 ± 2.17	7.87 ± 1.87	3.536	0.001
inferior septum, mm	9.37 ± 1.85	8.08 ± 1.81	3.977	<0.001
anterior septum, mm	10.48 ± 2.36	8.84 ± 1.87	5.218	<0.001
LVM(g)	101.18 ± 26.10	90.17 ± 25.86	2.965	0.007
LVMi(g/m^2^)	56.20 ± 13.01	50.01 ± 12.56	2.898	0.008
LV end-diastolic diameter, mm	52.67 ± 7.23	50.78 ± 6.94	2.517	0.017
Ventricular volume and systolic function				
Index LV end-diastolic volume, ml/m^2^	85.49 ± 15.27	78.45 ± 11.46	2.971	0.006
Index LV end-systolic volume, ml/m^2^	34.90 ± 8.28	30.17 ± 6.97	3.686	0.001
Index RV end-diastolic volume, ml/m^2^	85.13 ± 16.38	82.22 ± 16.25	0.694	0.496
Index RV end-systolic volume, ml/m^2^	37.21 ± 10.01	34.56 ± 11.11	1.274	0.217
LV ejection fraction, %	59.74 ± 5.76	61.70 ± 6.52	-1.689	0.105
RV ejection fraction, %	57.66 ± 7.03	59.27 ± 7.73	-1.223	0.233

Continuous variables with a normal distribution are presented as mean ± standard deviation. LV, left ventricular; RV, right ventricular; LVM, left ventricular mass; LVMi, left ventricular massindex; P < 0.05, P < 0.01 and P<0.001 indicate statistically significant differences between baseline parameters and posttreatment parameters.

A comprehensive evaluation of myocardial disease parameters for the 30 acromegaly patients was conducted, and the outcomes are detailed in **Table 6**. Post-treatment, T2 values in the basal (42.91 ± 4.38 *vs.* 39.12 ± 2.82ms, *P*<0.001) and apical (45.13 ± 4.87 *vs.* 41.96 ± 3.87ms, *P*=0.021) segments of the left ventricle exhibited a significant decrease compared to pre-treatment values. No significant differences were observed in left ventricular ECV values before and after treatment.

**Table 6:** Comparison of cardiomyopathic parameters in patients with acromegaly before and after 1 year of treatment.

Parameters	Baseline (n=30)	Posttreatment (n=30)	t	P
Myocardial Fibrosis				
Basal native T1, ms	1227.83 ± 35.76	1253.94 ± 53.86	-2.073	0.050
Basal post T1, ms	572.62 ± 60.70	493.70 ± 57.43	5.625	<0.001
Basal ECV, %	28.86 ± 2.97	29.05 ± 4.11	-0.188	0.853
Middle native T1, ms	1217.70 ± 52.68	1263.60 ± 53.20	-3.970	0.001
Middle post T1, ms	562.28 ± 61.57	487.31 ± 63.80	4.965	<0.001
Middle ECV, %	30.51 ± 4.16	29.80 ± 4.33	0.584	0.565
Apical native T1, ms	1242.86 ± 85.91	1272.02 ± 50.29	-1.480	0.154
Apical post T1, ms	551.48 ± 61.83	474.64 ± 61.95	5.251	<0.001
Apical ECV, %	32.91 ± 5.00	33.20 ± 4.31	-0.249	0.806
Myocardial Edema				
Basal T2, ms	42.91 ± 4.38	39.12 ± 2.82	4.733	<0.001
Middle T2, ms	43.05 ± 3.88	41.18 ± 3.53	1.956	0.062
Apical T2, ms	45.13 ± 4.87	41.96 ± 3.87	2.464	0.021

Continuous variables with a normal distribution are presented as mean ± standard deviation. ECV, extracellular volume; P < 0.05, P < 0.01 and P<0.001 indicate statistically significant differences between baseline parameters and posttreatment parameters.

### 3.4 The effect of biochemical remission on cardiac changes in acromegaly patients post-treatment

**Figure 2:**
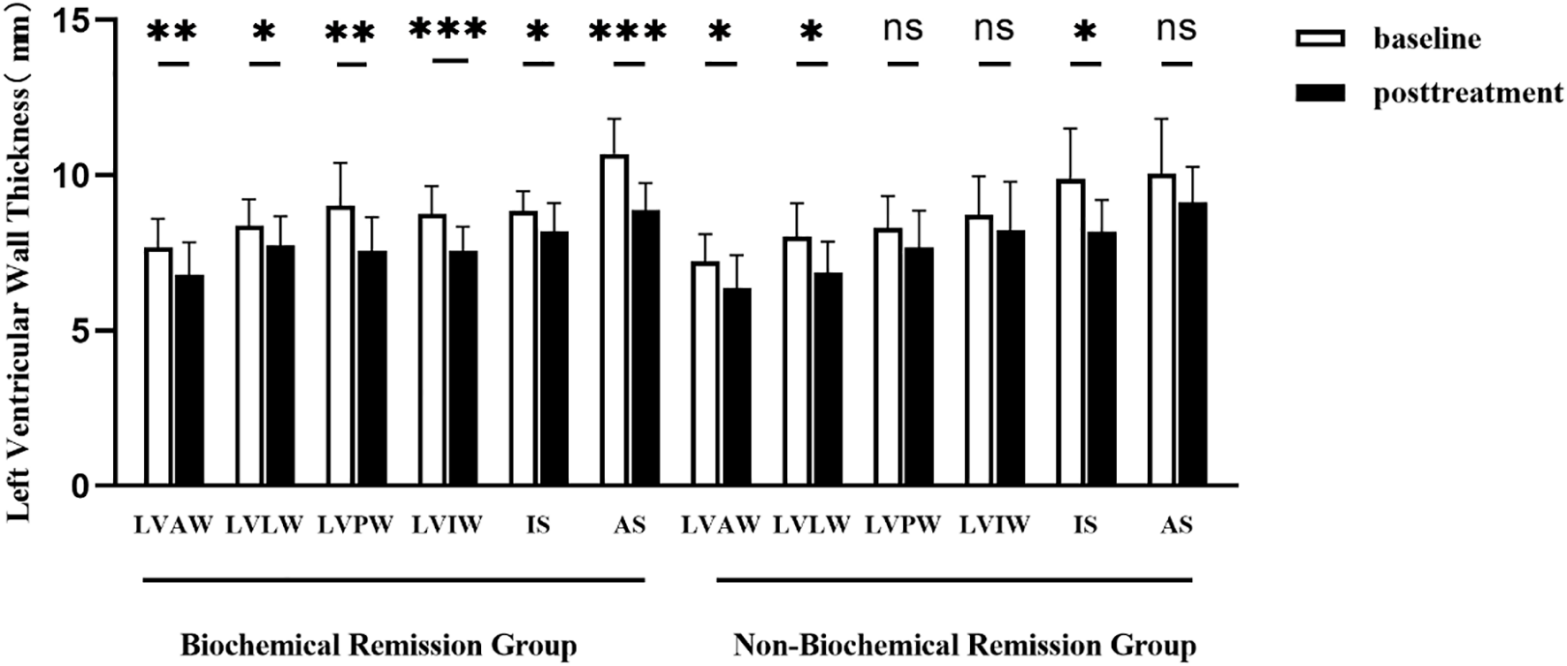
Comparison of left ventricular wall thickness before and after 1 year of treatment in acromegaly patients from the biochemical remission group and the non-biochemical remission group. LVAW, the left ventricular anterior wall; LVLW, left ventricular lateral wall; LVPW, left ventricular posterior wall; LVIW, left ventricular inferior wall; IS, inferior septum; AS, anterior septum. *(p<0.05), **(p<0.01) and ***(p<0.001) indicate significant differences. ns indicates no statistical difference.

Patients were stratified into the biochemical remission group (n=19) and non-biochemical remission group (n=11) based on their biochemical status after 1 year of treatment. The baseline clinical characteristics and cardiac parameters were similar in acromegaly patients with or without biochemical remission (**Table 7**). There were significant decrease in thickness of the left ventricular anterior wall, lateral wall, posterior wall, inferior wall, inferior septum, and anterior septum post-treatment in the biochemical remission group (**Figure 2**), with the most significant reductions in the left ventricular inferior wall (baseline 8.74 ± 1.89mm *vs.* post-treatment 7.58 ± 1.57mm, *P*=0.001) and anterior septum (baseline 10.69 ± 2.34mm *vs.* post-treatment 8.89 ± 1.81mm, *P*<0.001). In contrast, significant reduction was only observed in the thickness of the left ventricular anterior wall (baseline 7.23 ± 1.30mm *vs.* post-treatment 6.37 ± 1.58mm, *P*=0.014), lateral wall (baseline 8.02 ± 1.59mm *vs.* post-treatment 6.89 ± 1.46mm, *P*=0.005), and inferior septum (baseline 9.89 ± 2.40mm *vs*. post-treatment 8.17 ± 1.54mm, *P*=0.008) in the non-biochemical remission group (**Figure 2**). In addition, there were also significant decrease in left ventricular end-diastolic diameter (52.30 ± 5.17mm *vs.* 49.71 ± 4.97mm, *P*=0.009), left ventricular mass (105.27 ± 24.43g *vs.* 88.17 ± 25.80g, *P*=0.006) and left ventricular mass index (59.73 ± 12.18g/m^2^ *vs.* 48.79 ± 12.71g/m^2^, *P*=0.007) in the biochemical remission group, but not in the non-biochemical remission group (**Table 8**). There was significant decrease in T2 values in the basal, middle, and apical segments in the biochemical remission group, but only in the basal segment in the non-remission group (**Table 9**). However, the magnitudes of reduction one year post-treatment in all these Cardiac MRI parameters were not different between the biochemical and non-biochemical remission group. However, there was a trend toward smaller average left ventricular wall thickness, left ventricular mass, and T2 values in biochemical remission group (**Tables 8**, **9**).

**Table 7:** Comparison of baseline clinical characteristics between biochemical remission group and non-biochemical remission group.

Clinical characteristics	Biochemical remission group n=19	Non-biochemical remission group n=11	t/z	P
Age, years	40.74 ± 12.21	41.91 ± 12.14	-0.254	0.801
Male, n (%)	8 (42.1%)	3 (27.3%)	/	0.417
Tumor size, cm	2.04 ± 0.84	2.16 ± 1.16	-0.316	0.754
Disease duration, months	107.58 ± 82.51	121.27 ± 104.58	-0.397	0.694
Body mass index, kg/m^2^	25.99 ± 2.75	25.86 ± 4.81	0.084	0.934
Growth hormone nadir after the OGTT, ng/ml	10.70 (11.46, 38.34)	10.97 (3.78, 39.88)	0.294	0.771
insulin-like growth factor 1, ng/ml	482.59 ± 149.10	554.61 54170.64	-1.195	0.242
anterior pituitary insufficiency, n (%)	8 (42.1%)	8 (72.7%)	/	0.105

Continuous variables with a normal distribution are presented as mean ± standard deviation, while those with a non-normal distribution are expressed as median (interquartile range). Categorical variables are presented as percentages. OGTT, oral glucose tolerance test, P>0.05 indicate no statistically significant differences.

**Table 8:** Comparison of cardiac structural and functional parameters before and after 1 year of treatment in patients from the biochemical remission group and the non-biochemical remission group.

Parameters	Biochemical remission group, n=19			Non-biochemical remission group, n=11			P
	Baseline	posttreatment	change	Baseline	posttreatment	change	
Cardiovascular wall thickness and ventricular mass							
LV anterior wall, mm	7.67 ± 1.92	6.81 ± 2.15**	-0.86 ± 1.03	7.23 ± 1.30	6.37 ± 1.58*	-0.86 ± 0.96	0.989
LV lateral wall, mm	8.36 ± 1.78	7.75 ± 1.92*	-0.61 ± 1.21	8.02 ± 1.59	6.89 ± 1.46*	-1.14 ± 1.04	0.238
LV posterior wall, mm	9.01 ± 2.89	7.57 ± 2.22**	-1.44 ± 2.01	8.31 ± 1.53	7.67 ± 1.78	-0.64 ± 2.06	0.303
LV inferior wall, mm	8.74 ± 1.89	7.58 ± 1.57***	-1.16 ± 1.28	8.71 ± 1.86	8.21 ± 2.35	-0.50 ± 1.74	0.241
inferior septum, mm	8.86 ± 1.33	8.19 ± 1.90*	-0.67 ± 1.19	9.89 ± 2.40	8.17 ± 1.54*	-1.73 ± 1.75	0.058
anterior septum, mm	10.69 ± 2.34	8.89 ± 1.81***	-1.80 ± 1.59	10.06 ± 2.62	9.13 ± 1.72	-0.93 ± 1.58	0.157
LVM, g	105.27 ± 24.43	88.17 ± 25.80**	-17.10 ± 19.27	97.69 ± 20.41	94.28 ± 15.38	-3.41 ± 14.91	0.065
LVMi, g/m^2^	59.73 ± 12.18	48.79 ± 12.71**	-9.63 ± 11.33	55.01 ± 12.37	53.21 ± 10.06	-1.81 ± 8.21	0.067
LV end-diastolic diameter, mm	52.30 ± 5.17	49.71 ± 4.97**	-2.59 ± 3.87	51.14 ± 6.92	50.36 ± 5.95	-0.77 ± 4.84	0.267
Ventricular volume and systolic function							
LV end-diastolic volume, ml	157.90 ± 22.58	141.19 ± 23.63**	-16.71 ± 20.27	149.10 ± 41.31	142.00 ± 24.45	-7.10 ± 25.41	0.303
LV end-systolic volume, ml	64.24 ± 17.38	53.94 ± 16.63*	-10.29 ± 14.18	61.41 ± 20.30	55.68 ± 13.95	-5.74 ± 12.51	0.410
RV end-diastolic volume, ml	163.05 ± 32.60	148.62 ± 42.40	-14.43 ± 38.79	145.41 ± 37.66	146.55 ± 28.68	1.14 ± 27.03	0.270
RV end-systolic volume, ml	71.19 ± 22.16	61.04 ± 28.37	-10.15 ± 19.72	60.15 ± 17.87	62.58 ± 16.10	2.43 ± 7.26	0.057
LV ejection fraction, %	59.73 ± 6.93	62.30 ± 7.09	2.57 ± 7.92	59.23 ± 4.85	61.59 ± 5.68	2.37 ± 5.60	0.942
RV ejection fraction, %	57.07 ± 6.45	59.64 ± 9.79	2.57 ± 6.87	58.47 ± 8.27	58.39 ± 4.66	-0.08 ± 6.64	0.342

Continuous variables with a normal distribution are presented as mean ± standard deviation. LV, left ventricular; RV, right ventricular; LVM, left ventricular mass; LVMi, left ventricular massindex; *(p < 0.05), **(p < 0.01) and ***(p<0.001)indicate statistically significant differences between baselineparameters and posttreatment parameters. P indicates the differences in quantitative CMR parameter changes between the patients with and without biochemical remission at 1year after treatment.

**Table 9:** Comparison of myocardial disease parameters before and after 1 year of treatment in patients with acromegaly in the biochemical remission group and non-biochemical remission group.

Parameters	Biochemical remission group, n=19			Non-biochemical remission group, n=11			P
	Baseline	Posttreatment	Change	Baseline	Posttreatment	Change	
Myocardial Fibrosis							
Basal T1, ms	1232.64 ± 38.23	1259.60 ± 44.39*	26.96 ± 43.64	1225.28 ± 33.54	1230.43 ± 61.52	5.15 ± 78.79	0.401
Basal post T1, ms	565.93 ± 51.82	501.72 ± 40.47**	-64.21 ± 51.63	551.32 ± 76.69	486.54 ± 72.24*	-64.78 ± 65.45	0.982
Basal ECV, %	29.56 ± 2.99	29.78 ± 4.47	0.22 ± 4.89	28.57 ± 3.60	28.03 ± 3.11	-0.55 ± 4.18	0.680
Middle native T1, ms	1206.37 ± 67.37	1253.12 ± 43.78**	46.75 ± 53.13	1235.70 ± 21.14	1249.42 ± 26.92	13.72 ± 30.98	0.073
Middle post T1, ms	550.80 ± 54.42	496.75 ± 42.61**	-54.05 ± 57.53	548.74 ± 75.48	491.03 ± 80.62*	-57.71 ± 75.46	0.898
Middle ECV, %	30.76 ± 3.24	29.34 ± 3.14	-1.42 ± 4.77	30.32 ± 5.25	30.75 ± 5.45	0.43 ± 6.26	0.400
Apical native T1, ms	1230.92 ± 91.18	1279.54 ± 59.26	48.62 ± 91.91	1249.97 ± 48.21	1250.06 ± 47.17	0.09 ± 49.47	0.132
Apical post T1, ms	548.31 ± 45.90	487.76 ± 48.42**	-60.55 ± 54.93	531.61 ± 78.21	475.98 ± 77.34*	-55.63 ± 70.80	0.856
Apical ECV, %	31.76 ± 3.78	33.13 ± 4.62	1.47 ± 5.22	32.60 ± 6.01	31.92 ± 3.82	-0.68 ± 4.62	0.294
Myocardial Edema							
Basal T2, ms	41.32 ± 4.39	38.00 ± 3.42***	-3.32 ± 3.30	43.93 ± 4.67	39.87 ± 2.43*	-4.05 ± 4.99	0.629
Middle T2, ms	42.78 ± 3.60	39.60 ± 4.01**	-3.19 ± 4.66	42.79 ± 4.34	40.97 ± 3.75	-1.82 ± 4.52	0.440
Apical T2, ms	45.05 ± 4.81	41.36 ± 3.84*	-3.68 ± 6.40	45.47 ± 5.26	42.42 ± 3.68	-3.05 ± 6.17	0.795

Continuous variables with a normal distribution are presented as mean ± standard deviation. ECV, extracellular volume; *(P < 0.05), **(P < 0.01) and ***(P<0.001) indicate statistically significant differences. P indicates the differences in quantitative CMR parameter changes between the patients with and without biochemical remission at 1year after treatment.

### 3.5 Comparison of cardiac MRI parameters between biochemical and non-biochemical remission group and healthy control

Compared to healthy controls, patients with acromegaly in biochemical remission group one-year post-treatment exhibited greater left ventricular inferior septum thickness (8.18 ± 2.09mm *vs.* 5.83 ± 1.33mm, *P*=0.009) and higher extracellular volume (ECV) values for all myocardial layers of the left ventricle (all P<0.05)(**Table 10**). Additionally, the T2 value of the basal layer of the left ventricle was significantly lower in the biochemical remission group than in the healthy controls (38.59 ± 3.25ms *vs.* 41.99 ± 5.75ms, *P*=0.031). However, there were no significant differences in left ventricular mass, left ventricular mass index, or ejection fraction between the biochemical remission group and the healthy controls. In contrast, the non-biochemical remission group of acromegaly patients had significantly greater left ventricular inferior wall (8.48 8.2.29mm *vs.* 6.12 ± 1.19mm, *P*=0.019), inferior septum (8.25 8.1.60mm *vs.* 5.85 ± 1.86mm, *P*=0.019), anterior septum (9.41 9.1.53mm *vs.* 7.98 ± 1.33mm, *P*=0.043), and left ventricular mass (94.98 9416.03ms *vs.* 76.05 ± 20.39ms, *P*=0.025) compared to the healthy control group (**Table 11**). No significant differences in ventricular systolic function, ECV, and T2 values between the two groups.

**Table 10:** Comparison of cardiac MRI parameters between patients with acromegaly in biochemical remission group one-year post-treatment and healthy controls.

Parameters	Biochemical remission group n=16	Healthy control group n=16	t	P
Cardiovascular wall thickness and ventricular mass				
LV anterior wall, mm	6.72 ± 2.03	6.28 ± 1.50	0.639	0.533
LV lateral wall, mm	7.91 ± 2.07	6.49 ± 1.06	2.049	0.060
LV posterior wall, mm	7.62 ± 2.33	7.11 ± 1.40	0.622	0.544
LV inferior wall, mm	7.49 ± 1.72	6.25 ± 1.29	1.861	0.084
inferior septum, mm	8.18 ± 2.09	5.83 ± 1.33	3.014	0.009
anterior septum, mm	9.05 ± 2.00	8.01 ± 1.38	1.447	0.170
LVM (g)	97.64 ± 32.18	72.65 ± 20.20	2.115	0.054
LVMi (g/m^2^)	52.28 ± 15.29	40.22 ± 10.84	1.962	0.072
LV end-diastolic diameter, mm	49.73 ± 5.28	46.23 ± 6.09	1.461	0.166
Ventricular volume and systolic function				
LV end-diastolic volume, ml	143.05 ± 22.47	123.50 ± 37.07	1.408	0.184
LV end-systolic volume, ml	55.61 ± 17.71	49.12 ± 18.41	0.809	0.434
RV end-diastolic volume, ml	156.19 ± 55.07	107.07 ± 39.25	2.616	0.023
RV end-systolic volume, ml	69.92 ± 50.12	49.60 ± 18.71	1.355	0.200
LVEF (%)	61.66 ± 7.89	60.74 ± 3.40	0.374	0.715
RVEF (%)	58.50 ± 12.42	53.60 ± 6.54	1.339	0.205
Myocardial Fibrosis				
Basal T1, ms	1262.47 ± 41.82	1218.45 ± 58.54	2.229	0.050
Basal post T1, ms	504.83 ± 40.54	491.00 ± 33.42	0.675	0.515
Basal ECV, %	30.12 ± 3.66	26.19 ± 2.61	2.771	0.018
Middle native T1, ms	1258.19 ± 42.19	1210.23 ± 46.81	2.807	0.016
Middle post T1, ms	490.67 ± 46.32	484.54 ± 41.65	0.316	0.758
Middle ECV, %	29.53 ± 3.17	26.50 ± 3.01	3.012	0.010
Apical native T1, ms	1294.74 ± 43.46	1233.25 ± 91.32	2.461	0.032
Apical post T1, ms	475.03 ± 55.23	470.58 ± 50.36	0.175	0.864
Apical ECV, %	33.90 ± 4.48	28.87 ± 3.95	3.010	0.011
Myocardial Edema				
Basal T2, ms	38.59 ± 3.25	41.99 ± 5.75	-2.400	0.031
Middle T2, ms	39.82 ± 4.35	41.81 ± 3.14	-1.481	0.161
Apical T2, ms	41.42 ± 4.10	44.04 ± 5.47	-1.297	0.217

Continuous variables with a normal distribution are presented as mean ± standard deviation. LV, left ventricular; RV, right ventricular; LVM, left ventricular mass; LVMi, left ventricular massindex; LVEF, left ventricular ejection fraction; RVEF, right ventricular ejection fraction; ECV, extracellular volume; P < 0.05 indicate statistically significant differences between the biochemical remission group and the healthy control group.

**Table 11:** Comparison of cardiac MRI parameters between patients with acromegaly in non-biochemical remission group one-year post-treatment and healthy controls.

Parameters	Non-biochemical remission group n=10	Healthy control group n=10	t	P
Cardiovascular wall thickness and ventricular mass				
LV anterior wall, mm	6.53 ± 1.57	6.86 ± 1.69	-0.382	0.711
LV lateral wall, mm	7.04 ± 1.44	6.71 ± 1.23	0.545	0.599
LV posterior wall, mm	7.69 ± 1.87	7.39 ± 1.40	0.502	0.628
LV inferior wall, mm	8.48 ± 2.29	6.12 ± 1.19	2.845	0.019
inferior septum, mm	8.25 ± 1.60	5.85 ± 1.86	2.862	0.019
anterior septum, mm	9.41 ± 1.53	7.98 ± 1.33	2.349	0.043
LVM (g)	94.98 ± 16.03	76.05 ± 20.39	2.677	0.025
LVMi (g/m^2^)	52.71 ± 10.47	43.74 ± 12.21	1.994	0.077
LV end-diastolic diameter, mm	49.68 ± 5.80	46.42 ± 5.11	1.238	0.247
Ventricular volume and systolic function				
LV end-diastolic volume, ml	146.59 ± 21.49	127.82 ± 40.78	1.641	0.135
LV end-systolic volume, ml	58.19 ± 11.81	51.00 ± 20.17	1.196	0.262
RV end-diastolic volume, ml	151.62 ± 24.49	120.17 ± 52.03	1.729	0.118
RV end-systolic volume, ml	65.56 ± 13.39	57.49 ± 32.65	0.695	0.505
LVEF (%)	60.94 ± 5.53	60.61 ± 4.19	0.149	0.885
RVEF (%)	57.64 ± 4.17	53.59 ± 5.40	1.822	0.102
Myocardial Fibrosis				
Basal T1, ms	1247.87 ± 21.30	1215.33 ± 56.65	1.389	0.224
Basal post T1, ms	502.67 ± 91.03	498.83 ± 51.43	0.168	0.873
Basal ECV, %	28.34 ± 3.40	26.86 ± 3.19	0.720	0.495
Middle native T1, ms	1243.30 ± 24.26	1190.71 ± 37.37	2.730	0.034
Middle post T1, ms	484.33 ± 97.58	482.00 ± 54.74	0.086	0.935
Middle ECV, %	30.75 ± 5.45	27.25 ± 3.62	1.481	0.169
Apical native T1, ms	1235.71 ± 47.40	1218.57 ± 55.61	0.830	0.438
Apical post T1, ms	469.66 ± 87.82	466.14 ± 49.46	0.138	0.895
Apical ECV, %	31.92 ± 3.82	29.37 ± 3.68	1.426	0.184
Myocardial Edema				
Basal T2, ms	39.87 ± 2.43	40.64 ± 2.93	-0.620	0.549
Middle T2, ms	40.97 ± 3.75	40.34 ± 3.38	0.385	0.709
Apical T2, ms	42.42 ± 3.68	41.39 ± 4.76	0.570	0.581

Continuous variables with a normal distribution are presented as mean ± standard deviation. LV, left ventricular; RV, right ventricular; LVM, left ventricular mass; LVMi, left ventricular massindex; LVEF, left ventricular ejection fraction; RVEF, right ventricular ejection fraction; ECV, extracellular volume; P < 0.05 indicate statistically significant differences between the biochemical remission group and the healthy control group.

## 4 Discussion

Our findings revealed that Patients with acromegaly had left ventricular hypertrophy, left ventricular enlargement, myocardial fibrosis and decreased left ventricular systolic function assessed by Cardiac MRI compared with healthy controls. Treatment partially reversed these abnormalities, particularly in patients with biochemical remission. However, no improvement was observed in diffuse myocardial fibrosis, even in those with biochemical remission. Our study suggests that Cardiac MRI is a valuable tool for detecting early abnormalities and dynamic changes in cardiac structure and function in acromegaly patients before and after treatment.

In our study, the average left ventricular ejection (LVEF) in patients with acromegaly was lower than that of the healthy control group. Using an LVEF of less than 50% as the threshold for assessing impaired left ventricular systolic function (21, 22), we found that 5 patients had an LVEF below this threshold. Previous research results have been inconsistent. Guo et al.’s study suggested that compared to healthy individuals, patients with acromegaly showed an improvement in left ventricular systolic function (23). On the other hand, the study by Wolf et al. indicated a trend of decreased left ventricular ejection fraction when compared to the healthy control group (24). This may be related to the different stages of cardiac involvement in patients with acromegaly across various studies. Acromegaly-related cardiac disease is typically divided into three stages (25, 26). In the early stage, the heart exhibits concentric hypertrophy, but cardiac function remains unaffected, or may even be enhanced. In the intermediate stage, there is impairment of diastolic function at rest, and the left ventricular ejection fraction decreases during exercise. In the late stage, further structural damage occurs, leading to significant declines in both systolic and diastolic function, ultimately progressing to heart failure. In our study, the patients with acromegaly had a relatively long disease course, possibly indicating that they were in the intermediate to late stages of acromegaly-related cardiac disease.

LGE positivity was detected in 7 (17.9%) of 39 patients with acromegaly in this study, which indicates the presence of focal myocardial fibrosis in these patients. This is consistent with previous studies. A study in Brazil reported 5 cases (13.5%) exhibited LGE positivity using Cardiac MRI. Another study (13) showed 9 (14.8%) of 61 acromegaly patients exhibited LGE presence. The incidence of focal myocardial fibrosis was slightly higher than that was previously reported. Elevated GH levels (14.94 ng/ml) and longer duration of illness (median duration was 10 years) in our cohort may contribute to this increase. In the 7 acromegaly patients with focal myocardial fibrosis, the left ventricular ejection fraction was lower than that of the healthy control group. Among them, 5 patients had a left ventricular mass index higher than that of the control group, suggesting that myocardial hypertrophy and ventricular systolic dysfunction are, to some extent, related to the occurrence of myocardial fibrosis, similar to the findings of Guo et al.’s previous research (27). Furthermore, in our study, the ECV values in patients with acromegaly were higher than those in the healthy control group, indicating the presence of diffuse myocardial fibrosis. Previous studies have primarily used LGE imaging to assess myocardial fibrosis. However, for diffuse interstitial fibrosis, which affects a broader area but may be mild in severity, it can be challenging to visually distinguish the abnormal myocardial tissue from normal myocardium on MRI images. As a result, LGE imaging may have limitations in identifying such changes. In our study, we utilized both LGE imaging and T1 mapping techniques to evaluate myocardial fibrosis in patients with acromegaly, offering a more comprehensive depiction of myocardial involvement in these patients. Therefore, it is crucial to use T1 mapping technology to assess the presence of diffuse myocardial fibrosis in patients with acromegaly at an early stage. There was a trend toward increased extracellular volume (ECV) post-treatment, though it did not reach statistical significance. These results align with the previous Brazilian study, indicating that diffuse myocardial fibrosis in acromegaly patients may not be easily alleviated. However, studies with larger sample size are needed to validate this conclusion.

In our study, there was a trend of higher T2 values in the myocardial segments in the acromegaly group compared to that in the control group, but there were no significant differences between the groups. This indicates that patients with acromegaly tend to develop myocardial edema compared to healthy controls. This shows a slight discrepancy compared to previous research results. Gouya et al. utilized T2 mapping technology to assess myocardial edema in 15 patients with acromegaly and compared with 14 healthy volunteers (28). They observed higher T2 values in acromegaly patients compared to healthy volunteers before treatment. However, some studies have also indicated that there is no difference in T2 values between patients with acromegaly and the control group (24). It is well known that GH affects water balance, and that GH excess increases myocardial water content. The differences in research results may be related to varying levels of GH in acromegaly patients across different studies. In our study, the average GH level in acromegaly patients was significantly lower than that reported by Gouya et al.

In our study, acromegaly patients who achieved biochemical remission exhibited a decrease in T2 values in the basal, middle and apical segments of the left ventricle one year after treatment. Even among patients who did not achieve biochemical remission, there was a reduction in T2 values in the basal segment post-treatment, indicating a partial alleviation of myocardial edema. Despite not meeting the criteria for biochemical remission in terms of GH and IGF-1 levels, patients in the non-biochemical remission group showed a certain degree of reduction in both GH and IGF-1 levels compared to pre-treatment. This underscores the correlation between the decrease in T2 values and the reduction in GH and IGF-1 levels.

Although several cardiac parameters were found to be improved after one-year of treatment in biochemical and non-biochemical remission group, there were no significant differences in all the Cardiac MRI parameters between them. Only a trend toward lower average left ventricular wall thickness, left ventricular mass, and T2 values in the biochemical remission group compared to the non-biochemical remission group. This may be related to the small sample size of our study, and further research with larger sample sizes is needed to validate these findings.

In our study, no significant differences were observed in left ventricular wall thickness, left ventricular mass index, ventricular volumes, T2 values, or extracellular volume fraction between patients with acromegaly—regardless of whether biochemical remission was achieved—and healthy controls. A multicenter case-control study published in 2024 (29) reported findings similar to ours, demonstrating that ventricular structural and functional abnormalities may persist in patients with acromegaly even after achieving biochemical control.

However, our study not only evaluated the differences in cardiac parameters between treated acromegaly patients and healthy controls, but also compared cardiac parameters in patients with acromegaly before and one year after treatment. Our findings suggest that, in patients who achieved biochemical remission, there were significant reductions in left ventricular wall thickness, left ventricular mass index, left ventricular end-systolic volume, left ventricular end-diastolic volume, and T2 values after one year of treatment, whereas no significant change was observed in extracellular volume fraction (ECV). These results indicate that the structural and functional abnormalities of the heart in acromegaly may be partially reversible following treatment; however, myocardial fibrosis may be less amenable to reversal. Nonetheless, it cannot be ruled out that with extended follow-up periods and biochemical control, myocardial fibrosis may improve. Therefore, future studies with larger sample sizes and longer follow-up durations are needed to verify these findings.

## 5 Conclusion

In this prospective cohort study, we employed Cardiac MRI to evaluate cardiac structure, function, and myocardial tissue characteristics in acromegaly patients before and one year after treatment, aiming to elucidate the influence of biochemical remission on cardiac alterations in this population. The findings indicate that in patients with acromegaly who have undergone successful treatment and achieved biochemical remission, there is a certain degree of restoration in cardiac structure and function, but no significant improvement in myocardial fibrosis. This study emphasizes the accuracy of cardiac magnetic resonance imaging in assessing cardiac structure and function in acromegaly patients, offering valuable insights for early intervention in cardiac complications and potentially enhancing long-term quality of life and survival rates for this patient cohort.

## 6 Strengths and limitations

This study not only compared the cardiac parameters of patients with acromegaly to those of a healthy control group using cardiac magnetic resonance imaging but also compared the cardiac parameters of acromegaly patients before and after treatment. Additionally, our research provided a comprehensive assessment of myocardial fibrosis in acromegaly patients using LGE imaging and T1 mapping techniques. However, our study also has some limitations. Firstly, this study is a single-center cohort study. Given the relative rarity of acromegaly, the sample size is relatively limited, necessitating further research efforts to enlarge the sample and enhance the study’s generalizability. Secondly, the study predominantly employed Cardiac MRI technology to appraise cardiac structure and functional status in acromegaly patients, with a specific emphasis on myocardial involvement. Notably, other cardiac complications, including arrhythmias and valve damage, were not systematically evaluated. Consequently, the assessment of cardiac complications in acromegaly may lack comprehensiveness, and future investigations should consider a more holistic approach to encompass a broader spectrum of potential cardiac issues. Thirdly, in our study, treatment for patients with acromegaly included either monotherapy or combination therapy. However, due to the limited number of patients in certain subgroups, we did not perform an analysis of the impact of different treatment modalities on cardiac magnetic resonance imaging parameters in patients with acromegaly.

## Data Availability

The raw data supporting the conclusions of this article will be made available by the authors, without undue reservation.
